# Proteinuria in preterm neonates: influence of fetal growth restriction

**DOI:** 10.1038/s41372-025-02306-0

**Published:** 2025-04-19

**Authors:** Arvind Sehgal, Criona Levins, Emma Yeomans, Zhong Lu, David Metz

**Affiliations:** 1 Monash Newborn, Monash Children’s Hospital, Melbourne, VIC Australia; 2 Department of Pediatrics, Monash University, Melbourne, VIC Australia; 3 Department of Biochemistry, Monash Health, Melbourne, VIC Australia; 4 Department of Nephrology, Royal Children’s Hospital, Melbourne, VIC Australia

**Keywords:** Kidney diseases, Outcomes research

## Abstract

**Objective:**

To compare proteinuria in preterm neonates with fetal growth restriction-small for gestational age (FGR-SGA) against equally preterm but appropriate for gestational age (AGA) neonates.

**Study design:**

Prospective, observational cohort study.

**Results:**

Eighteen FGR-SGA neonates were compared with 18 AGA neonates (gestation; 29 ± 1 vs 29 ± 2 weeks, *P* = 0.8). Urine total protein (median [interquartile range]) in FGR-SGA was higher 370 [323, 573] vs 255 [193, 453] mg/L in AGA, *P* = 0.017 at first assessment (week one) and 565 [445, 743] vs 225 [135, 458] mg/L, *P* = 0.0011 at second assessment (week four). Urine protein creatinine ratio was 393 [250, 445] in FGR-SGA vs 227 [163, 367] mg/mmol in AGA, *P* = 0.029 at first assessment and 444 [368, 699] vs 240 [199, 411] mg/mmol, *P* = 0.0014 at second assessment. Mean blood pressure was higher in FGR-SGA group & directly correlated with proteinuria.

**Conclusions:**

Increased proteinuria in FGR-SGA suggests reduced nephron endowment and hyper-filtration.

## Introduction

Premature birth increases the risk of chronic kidney disease (CKD) in later life, with greater prematurity associated with greater risk. Because nephrogenesis continues until 36 weeks gestational age (GA), neonates born prematurely suffer from disturbed nephrogenesis leading to possible reduced nephron endowment, and less glomeruli compared to those born at term [1]. Recently, consensus guidelines for monitoring of kidney health in ‘NICU graduates’ have been published, which suggest longitudinal surveillance for those at risk [2]. With increased survival of premature neonates, close to 2 million neonates delivered <32 weeks GA may be affected [3]. A more granular risk-stratification is desirable [2]. Various factors can increase the risk of CKD in ex-premature neonates; including fetal growth restriction (FGR), which complicates a large number of preterm deliveries (18–27%) [4, 5]. A meta-analysis including >2 million individuals found that a history of low birthweight was associated with an 80% increased likelihood of albuminuria after 12 months of age, and a strong association with subsequent risk of CKD [6]. A recent study compared 25 FGR-SGA neonates at median GA 33 weeks (range 31–36) with appropriate for gestational age (AGA) neonates (38 [35–41]) weeks, at 18 months of age. Urinary albumin-urine creatinine ratio was significantly higher in the FGR-SGA subjects [7].

We hypothesized that in light of previous experimental and clinical data, the magnitude of proteinuria may be higher in preterm-FGR-SGA neonates during early postnatal period. The objectives of this study were to ascertain proteinuria in a cohort of premature FGR-SGA neonates, compared with equally premature AGA neonates.

## Methods

This prospective, observational cohort study was conducted over approximately 2-years (July 2022 to March 2024) at a quaternary neonatal intensive care unit. As an exploratory, hypothesis generating study, neonates were enroled opportunistically over the time period without formal a priori power calculation. FGR-SGA was defined as birthweight <10^th^ percentile for GA and sex, and absent/reversed antenatal Doppler in fetal systemic arteries [4, 8, 9]. Neonates with known renal or congenital cardiac abnormalities or FGR due to genetic causes were excluded. Potential participants were flagged on admission based on inclusion and exclusion criteria and investigator availability. Inclusion criteria for the FGR-SGA cohort was GA < 32 weeks and birthweight <10^th^ percentile for GA and sex, and Doppler abnormalities. They were compared with AGA neonates of comparable gestation, not necessarily subsequent or consecutive births (not in tandem but random). The latter group had no large for gestational age neonates. GA and birthweight were the only inclusion criteria. A spot bag urine sample was collected at two time points (end of first week and repeated at four weeks of age) and sent for testing following collection. Samples contaminated with stool were discarded and repeated. Serum protein and creatinine were assayed coinciding with urine collections. We used proteinuria and urine protein creatinine ratio (UPCR) as surrogates for hyper-filtration mediated kidney damage. Urine total protein levels were expressed relative to creatinine levels (UPCR). Urine total protein level was measured by Pyrogallol Red Colorimetry method. For urine creatinine level, we used the IDMS traceable Jaffe’ kinetic method. Serum protein was measured by Biuret Cupric ion Colorimetric method and serum albumin was measured by Bromo Cresol Purple (BCP) Colorimetric method. All the tests were analysed using an AU5800 automated Chemistry analyser (Beckman Coulter, Brea, USA).

Clinical data such as concomitant therapy including nephrotoxins, any inotropic use and respiratory support were recorded. Pathological proteinuria was defined as urine protein concentration ≥500 mg/L [10]. Acute kidney injury (AKI) was defined based on neonatal oliguria per neonatal KDIGO criteria [11], or critical value thresholds of serum creatinine [12, 13]. In addition, we compared serum creatinine values to gestation- and post-natal age equivalent normative data [14]. The duration of antibiotics in the Unit is guided by culture positivity. Paracetamol (and not indomethacin/ibuprofen) is used for ductal closure. The Unit does not practice intraventricular haemorrhage prophylaxis with indomethacin.

A single daily morning non-invasive blood pressure (BP) reading was recorded in quiet awake state with appropriate size cuff using Drager Infinity (Drägerwerk AG & Co. KGaA Moislinger Allee 53–55 23558 Lübeck, Germany).

### Statistics

Descriptive statistics were used to characterize baseline information. Neonate characteristics and outcomes were compared using Student’s *t* test for parametric continuous variables, Mann–Whitney U test for non-parametric continuous variables, and chi-square or Fisher’s exact test for categorical variables. Pearson correlation was used to test associations between BP and urine parameters. Two-tailed significance was set at *P* < 0.05. Analyses were performed using Stata software BE/17 and R version 4.4.1 (2024).

## Results

Thirty-six neonates formed the study cohort; the GA at birth between the two groups was comparable (29 ± 1 vs 29 ± 2 weeks, *P* = 0.8). Table 1 depicts demographic variables. The GA and day of life at the first and second urine assessments were comparable. Number of male neonates in both groups were comparable. None of the neonates were on antibiotics, inotropes, or paracetamol at either time points. Neonates with suspected late onset sepsis are tested for UTI; none had documented UTIs. No neonates in this cohort had umbilical artery catheter.

**Table 1:** Demographic and proteinuria information of the study cohort.

Variable	FGR-SGA (*n* = 18)	AGA (*n* = 18)	*P*
First assessment			
GA at birth (weeks)	29 ± 1	29 ± 2	0.8
Birthweight (g)	801 ± 240	1148 ± 259	0.0002
GA@ sample (weeks)	29 ± 2	29 ± 2	0.7
Weight at sample (g)	801 ± 254	1112 ± 246	0.0009
Day of life	5 ± 2	5 ± 2	0.8
Systolic BP (mm Hg)	57 ± 10	60 ± 10	0.3
Mean BP (mm Hg)	42 ± 7	44 ± 6	0.1
Male sex *n* (%)	10 (55)	8 (44)	0.7
Antenatal steroids *n* (%)	12 (67)	14 (78)	0.7
Surfactant *n* (%)	8 (44)	10 (55)	0.7
Mode of delivery (Caesarean section) *n* (%)	16 (89)	14 (78)	0.4
CPAP *n* (%)	14 (78)	10 (55)	0.3
Urine total protein (mg/L)	370 (317, 573)^a^	255 (193, 453)^a^	0.017
Urine creatinine (mmol/L)	1.27 ± 0.6	1.17 ± 0.4	0.6
Urine protein: creatinine ratio (mg/mmol)	393 (250, 445)^a^	227 (163, 367)^a^	0.029
Serum protein (g/L)	50.2 ± 5	47.3 ± 3.7	0.2
Serum creatinine (µmol/L)	58 ± 10	61 ± 11	0.4
Second assessment			
Weight at sample (g)	1048 ± 359	1494 ± 397	0.001
Day of life	23 ± 6	25 ± 8	0.5
Systolic BP (mm Hg)	68 ± 10	61 ± 6	0.02
Mean BP (mm Hg)	49 ± 7	42 ± 4	0.01
CPAP *n* (%)	12 (67)	7 (40)	0.2
Urine total protein (mg/L)	565 (445, 743)^a^	225 (135, 458)^a^	0.004
Urine creatinine (mmol/L)	1.2 ± 0.4	1.02 ± 0.5	0.2
Urine protein: creatinine ratio (mg/mmol)	444 (368, 699)^a^	240 (199, 411)^a^	0.003
Serum protein (g/L)	47.2 ± 5	45.1 ± 5	0.4
Serum creatinine (µmol/L)	56 ± 8	60 ± 9	0.1

*BP* blood pressure, *GA* gestational age, *CPAP* continuous positive airway pressure, *FGR* fetal growth restriction, *AGA* appropriate for gestational age, FGR-SGA-defined as birthweight <10^th^ percentile for GA and sex, and absent/reversed antenatal Doppler in fetal systemic arteries [4, 8, 9].

^a^Median (interquartile range).

There were no episodes of AKI in either cohort, either based on neonatal oliguria or on critical value thresholds for serum creatinine. All serum creatinine values were within the 95^th^ percentile for gestation and post-natal age at first sampling occasion, with 2/36 and 3/36 above the 95^th^ percentile ranges at sampling occasion 2. There was no significant difference in serum or urine creatinine values between cohorts at either sample occasion (see Table 1).

**Fig. 1:**
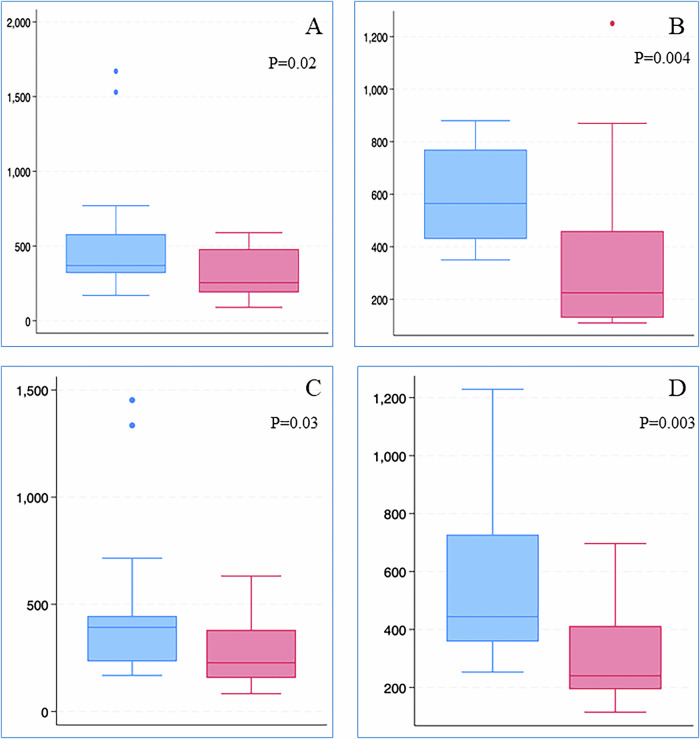
Comparison of Urine total protein and Urine protein/creatinine ratios between fetal growth restriction (blue) and appropriate for gestational age (red) neonates. **A** Urine total protein (mg/L) (assessment 1). **B** Urine total protein (mg/L) (assessment 2). **C** Urine protein creatinine ratio (mg/mmol) (assessment 1). **D** Urine protein creatinine ratio (mg/mmol) (assessment 2).

**Fig. 2:**
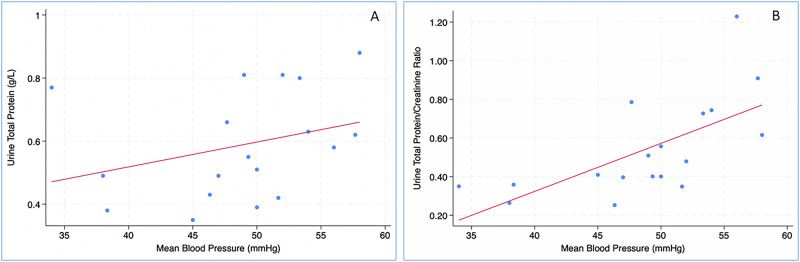
Influence of blood pressure on proteinuria. Correlation between mean blood pressure and urine total protein (**A**) and urine total protein creatinine ratio (**B**) in neonates with fetal growth restriction (FGR) at second assessment.

Urine protein concentration and UPCR were significantly higher in FGR-SGA vs AGA neonates at both time points (Table 1 and Fig. 1). Proteinuria ≥500 mg/L was noted in 7/18 (39%) FGR-SGA neonates (vs 3/18 [17%] AGA neonates) at first assessment (*P* = 0.2), and 11/18 (61%) FGR-SGA neonates (vs 2/18 [11%] AGA neonates) at second assessment (*P* = 0.004). Mean BP was significantly higher in the FGR-SGA group at second urine assessment (*n* = 18, 49 ± 7 vs 42 ± 4, *P* = 0.01) (Table 1). A significant increase in mean and systolic BP was noted amongst FGR-SGA neonates between assessments one and two (*P* = 0.009 and *P* = 0.002, respectively). In comparison, the change in AGA cohort was not significant (mean BP, *P* = 0.2 and systolic BP, *P* = 0.7, respectively). Importantly, the mean BP at the second assessment correlated significantly with urine protein (*n* = 18, r^2^ = 0.4, *P* = 0.004) and UPCR (*n* = 18, r^2^ = 0.43, *P* = 0.003) in FGR-SGA neonates (Fig. 2); but not in AGA neonates (r^2^ = 0.02, *P* = 0.6 and r^2^ = 0.07, *P* = 0.3, respectively). From assessment one to assessment two, the change in levels of urine protein or UPCR (*n* = 18) was statistically non-significant in either FGR-SGA or AGA group.

## Discussion

In this prospective observational study, we describe for the first time significantly higher proteinuria in the first month of life in FGR-SGA neonates, as compared to AGA neonates of equivalent gestational age. This was shown without influence of identifiable haemodynamic or inflammatory confounding at either time point. Increased proteinuria was apparent in the first week and persisted at one month of age. We additionally compared magnitude of proteinuria in the FGR-SGA cohort with that of published normative data (Supplementary Table S1) [15–17]. Whilst proteinuria in the AGA group was equivalent with literature values, the FGR-SGA group had increased proteinuria compared with equivalently premature neonates. Finally, this proteinuria correlated with higher BP in the FGR-SGA neonates.

Our results provide evidence supportive of early glomerular hyper-filtration (elevated BP and increased proteinuria) in FGR-SGA neonates. This is supportive of the hypothesis that utero-placental insufficiency and the accompanying blood-flow redistribution could impact renal function, adding to the emerging evidence of proteinuria in older age-groups who were preterm and FGR-SGA at birth [18, 19], as well as increased prevalence of CKD in later childhood and adults. Pathophysiologically, this phenomena is due to reduced nephron number and filtration surface, with subsequent glomerular hyper-filtration to maintain equivalent clearance. This comes at the cost of glomerular hypertension, with proteinuria and glomerular injury over time and, if not ameliorated, CKD.

### Knowledge gaps: contextual to previous literature

In pregnancies with fetal growth restriction, cerebral redistribution and diversion of blood away from organs considered less vital (such as kidneys), reflects in abnormal antenatal Doppler recordings. This leads to irreversible changes to metabolism and organ functioning, with possible detrimental effects in later life [20, 21]. Information from both experimental and human studies informs that FGR-SGA impacts kidney volume and nephron number [18, 22–24]. Reduced filtration surface area in affected kidneys could predispose to glomerular and systemic hypertension, glomerular damage and sclerosis, adversely affecting renal function via the ‘hyper-filtration theory’ [25]. Our study noted higher urinary proteinuria in FGR-SGA cohort, highlighting the effects of peculiarities pertinent to FGR-SGA and aims to address an apparent knowledge gap in pre-existing literature. Proteinuria is typically assessed using the UPCR, which controls for urinary concentration and is less cumbersome than the gold-standard 24 h urinary protein excretion. Normative data is accumulating for term and preterm neonates (Supplementary Table S1), though this is the first study comparing FGR-SGA with AGA neonates of comparable gestational age. Comparable gestational age of controls is critical in assessing impact of FGR-SGA, given proteinuria increases with increasing degree of prematurity.

A recent study grouped neonates according to GA (≤28 weeks, 29–31 weeks, 32–36 weeks and ≥37 weeks) and performed urine assessments at weekly intervals from first week until day 28. Urine protein was inversely associated with GA at birth, however, no change was noted with increasing postnatal age [10]. This study also defined pathological proteinuria as urine protein concentration ≥500 mg/L, though defining a threshold based on protein concentration risks bias by not accounting for urine volume and concentration. Within this limitation, they observed it in 12 (9.3%) neonates at one or more time points, majority belonging to ≤28 weeks GA. This study did not analyse FGR-SGA neonates separately. In our study, pathological proteinuria was more common in FGR-SGA neonates. Another study analysed urine samples from 231 preterm neonates within the first 48 h and/or between 72–120 h of life [15]. Only 16 (7%) samples were from very preterm neonates; median (interquartile range) UPCR was 214 (172, 261) mg/mmol, which reduced with increasing GA from <29 weeks to 37 weeks. Again, FGR neonates were not analysed separately. Similarly, amongst neonates <30 weeks, 30–36 weeks and >36 weeks during the first week of life, while UPCR significantly declined with increase in GA, no information about FGR status was provided [26].

### Mechanistic links between FGR-nephron endowment-proteinuria

A causal relationship between FGR-SGA and impaired nephrogenesis is supported by experimental models as well as autopsy studies in human FGR cohorts [18, 24, 27–30]. This association may increase the risk of renal failure and end-stage renal disease in later life [31, 32].

**Fig. 3:**
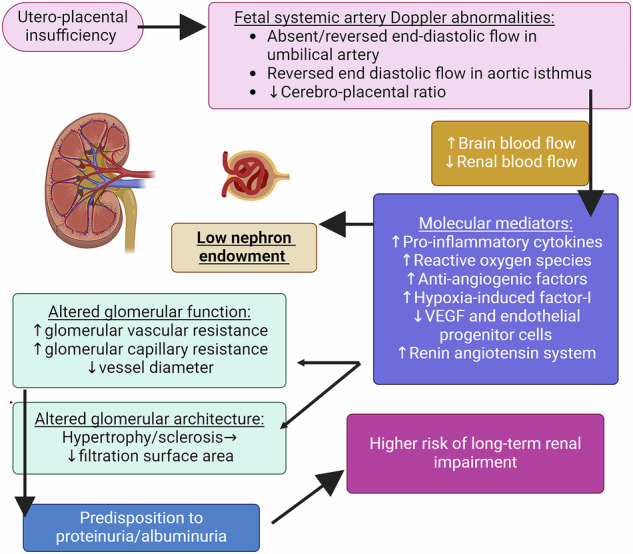
Mechanistic links and molecular mediators between utero-placental insufficiency and urine protein leakage. VEGF vascular endothelial growth factor. Created on Biorender.

Figure 3 summarizes mechanistic links and molecular mediators between utero-placental insufficiency and urine protein leakage; putatively mediated through abnormal fetal organ perfusion [33–39]. The compensatory humoral mechanisms include the activation of the renin-angiotensin system (RAS) in an attempt to increase glomerular filtration rate. Cord blood samples from FGR-SGA births have previously indicated heightened RAS [40], especially when Doppler flow abnormalities are noted antenatally [41, 42]. Causative role of RAS and elastin degradation (and its replacement by collagen) in hypertension in FGR cohorts has been recently summarised by us and other investigators [43–45].

### Interlinking hypertension-arterial stiffness-renal dysfunction in FGR cohorts

We noted significantly higher BP in FGR-SGA neonates, which correlated with proteinuria; this was not the case in AGA neonates. Higher BP and arterial stiffness have been previously noted in FGR-SGA neonates in the first few weeks of life as well as across age-groups [46–49]. Conduit arteries cushion ventricular waveforms but this well-known ‘windkessel effect’ is lost due to arterial stiffness in FGR cohorts, transmitting high-pressure to a normally high-flow, low-resistance circulation (such as glomeruli), damaging the fragile microvasculature. Microvascular endothelial dysfunction and reduced vascular diameter superimposed on reduced nephron numbers leads to increased glomerular vascular resistance [40, 50, 51]. Hence, a reduced capability of afferent renal arterioles to accommodate the undamped arterial waveform results in compensatory glomerulomegaly, hyper-filtration, and proteinuria via heightened activation of RAS [52]. Ameliorating this, for example with RAS inhibition, could be of therapeutic benefit.

Experimental data in rats and lambs indicate that angiotensin converting enzyme (ACE) inhibition for a brief period early in postnatal life can prevent urine protein loss and result in higher renal blood flow with lower filtration fraction; benefits persisting much beyond treatment withdrawal [53, 54]. In premature neonates (<35–36 weeks GA), calcium channel blockers are generally preferred to manage systemic hypertension. As per Unit practice, preterm neonates <37 weeks corrected gestational age are not administered ACE inhibitors because of impaired nephrogenesis risk [55]. For older neonates, our preference is for ACE inhibition due to its physiological superiority, especially considering endothelial and arterial extra-cellular matrix effects of ACE inhibition which might be contributory to renal microvascular injury (and consequent urine protein loss).

### FGR and long-term renal impairments: population data

The findings of our study should be interpreted in the context of pre-existing population data which indicate FGR-SGA cohort to be at higher risk for developing renal disease [56]. An Australian study of aboriginal subjects between 4 and 72 years of age showed that those with the lowest birthweight had the smallest kidneys, the highest BP and the highest rates of urine protein loss [57]. Similarly, a Canadian study noted that subjects with end-stage renal disease were three times more likely to have had low birthweight compared with healthy subjects [31]. A prospective follow-up study of >400 subjects with mean age of 19 years who were born very preterm, determined that FGR state predisposed to urine protein loss [58]. Lastly, in a Norwegian study of subjects between 18 and 42 years of age, birthweight <10^th^ percentile for GA was significantly associated with the risk of end-stage renal disease; the effect being strongest in those born preterm and FGR-SGA [59]. It appears that FGR-SGA is a risk factor for development of progressive renal disease, and pediatric and adult renal physicians should routinely explore history of prematurity and FGR in patient populations.

### Follow up and implications for future research

Findings of this study should motivate structured longitudinal renal follow-up of high-risk neonates, with the addition of FGR-SGA as potential risk factor for early proteinuria. Long-term strategies include BP surveillance and proteinuria screening, allowing for early institution of nephroprotective strategies including anti-proteinuric therapies, should parameters progress to treatment thresholds. The first week of life sees substantial variability in urinary creatinine concentration due to differences in maternal and early serum creatinine, and increasing renal blood flow. This leads to increased variability of the UPCR due to variability in denominator [60]. Nevertheless, normative data from a number of cohorts of relatively large size, with 95^th^ percentile thresholds, help to define clear outliers, despite this additional noise; our FGR-SGA cohort exceeding these limits. We also note largely equivalent serum and urine creatinine between cohorts, likely minimising such impact. Finally, and crucially, whilst this greater variability in urine creatinine is applicable in the 1^st^ week of life, the significant difference in proteinuria between FGR-SGA and AGA cohorts remained at second assessment.

Limitations of this exploratory study include relatively small numbers and short-term outcome. Neonates are routinely transferred to low-acuity neonatal units for ongoing care closer to home. We did not measure other proteins in urine like albumin, β2 urine macroglobulin or retinol binding protein (laboratory limitations). We thus cannot rule out a contribution from tubular injury or immaturity. Finally, we note the equivalent UPCR in our AGA cohort with published normative data, as compared to UPCR in FGR-SGA cohort which showed significantly higher UPCR than AGA cohort.

## Conclusions

We identified increased proteinuria in preterm FGR-SGA neonates, when compared with equally preterm but AGA neonates, as well as population normative data. This adds to evidence of premature FGR-SGA neonates being at greater risk of reduced nephron endowment, with early hyper-filtration, and that UPCR might be used to identify neonates requiring longitudinal renal surveillance.

## Supplementary information

Table

## Data Availability

The datasets generated during and/or analysed during the current study are available from the corresponding author on reasonable request.
