# Supplementary material for: Proteinuria in preterm neonates: influence of fetal growth restriction
Source: J Perinatol. 2025 Apr 19;45(10):1469–75. doi: 10.1038/s41372-025-02306-0 (PMC12479344; doi:10.1038/s41372-025-02306-0)
Supplement: Supplementary file 1 — Table [file 41372_2025_2306_MOESM1_ESM.docx]

Supplementary table S1. Summary of published data on preterm neonates for urine protein creatinine ratio (UPCR), urine albumin creatinine ratio (UACR)

| **UPCR** | **WOL1** | | | **WOL2** | **WOL3** | **WOL4** |
| --- | --- | --- | --- | --- | --- | --- |
|  | **Day 0-1** | **Day 3-4** | **Day 5-6** |  |  |  |
| FGR group^  (26-32/40 weeks) |  |  | 380 (222, 445) |  |  | 409 (358, 727) |
| AGA group^  (26-32/40 weeks) |  |  | 227 (155, 383) |  |  | 240 (192, 428) |
| Ref (17)  (<32/40 weeks) | 191 (114–399) | 226 (152–319) | 225 (156–350) | 282 (200–488) | 308 (188–576) | 325 (175–664) |
| Ref (16)  (<29/40 weeks) | 149 (105–227) | 214 (172–261) |  |  |  |  |
| Ref (16)  (29-33/40 weeks) | 108 (81–140) | 130 (102–147) |  |  |  |  |
| Ref (16)  (>33/40 weeks) | 61 (46–90) | 93 (71–123) |  |  |  |  |
| Ref (18)  (Term)* | 62 (24-162) | 65 (4-136) |  |  |  |  |
| **UACR** | **WOL1** | | | **WOL2** | **WOL3** | **WOL4** |
|  | **Day 0-1** | **Day 3-4** | **Day 5-6** |  |  |  |
| FGR group^  (26-32/40 weeks) |  |  | 55 (35, 110) |  |  | 54 (23, 90) |
| AGA group^  (26-32/40 weeks) |  |  | 31 (16, 55) |  |  | 32 (23, 51) |
| Ref (16)  (<29/40 weeks) | 22 (15–48) | 50 (35–57) |  |  |  |  |
| Ref (16)  (29-33/40 weeks) | 21 (12–42) | 21 (14–29) |  |  |  |  |
| Ref (16)  (>33/40 weeks) | 8 (5–14) | 12 (6–19) |  |  |  |  |
| Ref (18)  (Term)* | 14 (2, 84) | 8 (0,34) |  |  |  |  |

Values in median (interquartile range), *median (minimum-95^th^ percentile), FGR-fetal growth restriction, AGA- appropriate for gestational age, WOL-week of life, ^current study
